# Costs of multidrug-resistant TB treatment in Finland and Estonia affected by the 2019 WHO guidelines

**DOI:** 10.5588/ijtld.20.0892

**Published:** 2021-07-01

**Authors:** T. Feuth, R-L. Patovirta, S. Grierson, M. Danilovits, P. Viiklepp, H. K. Aaltonen, M. Vauhkonen, L. Pehme, T. Vasankari

**Affiliations:** 1 Department of Pulmonary Diseases and Clinical Allergology, University of Turku, Turku; 2 Division of Medicine, Department of Pulmonary Diseases, Turku University Hospital, Turku; 3 Department of Respiratory Medicine, Kuopio University Hospital, Kuopio; 4 Finnish Lung Health Association (FILHA), Helsinki, Finland; 5 Lung Clinic of Tartu University Hospital, Tartu, Estonia; 6 National Institute for Health Development, Tallinn, Estonia

**Keywords:** multidrug-resistant tuberculosis, tuberculosis, bedaquiline

## Abstract

**BACKGROUND:**

Multidrug-resistant TB (MDR-TB) is a growing problem in the effort to end the global TB epidemic. In 2019, the WHO adopted a new standardised regiment for MDR-TB, consisting of only oral medications.

**METHODS:**

We estimated the impact of the new guidelines on the costs of TB treatment in Estonia and Finland. For both countries, the costs of the two most common new drug regimens were calculated, including drug costs, as well as care- and monitoring-related costs.

**RESULTS:**

In Turku, Finland, treatment costs with the old regimen were €178,714; this could either increase by 10% or decrease by 18%, depending on the duration of bedaquiline use (6 months vs. 20 months). In Estonia, treatment costs with the old regimen were €33,664, whereas the new regimens were associated with a 40% increase in overall costs.

**CONCLUSIONS:**

The 2019 WHO guidelines have led to significant changes in the costs of MDR-TB treatment in Finland and Estonia. These changes depend mostly on the drug regimen administered and on care-related practices, with important differences between countries and even within the same country due to local practices.

## Abstract (FR)

**CONTEXTE:**

La TB multirésistante (MDR-TB) constitue un problème croissant dans les efforts de mettre fin à l’épidémie mondiale de TB. En 2019, l’OMS a adopté un nouveau protocole standardisé pour la MDR-TB, consistant seulement en médicaments oraux.

**MÉTHODES:**

Nous avons estimé l’impact des nouvelles directives sur le coût du traitement de la TB en Estonie et en Finlande. Dans ces deux pays, les coûts des deux nouveaux protocoles les plus fréquents ont été calculés, y compris le coût des médicaments ainsi que celui de la prise en charge et du suivi.

**RÉSULTATS:**

A Turku, Finlande, les coûts ont été de 178 714€ avec le protocole ancien et ils pourraient augmenter de 10% ou diminuer de 18% selon la durée d’administration de la bédaquiline (6 mois contre 20 mois). En Estonie, les coûts du protocole ancien étaient de 30 131€, tandis que les nouveaux protocoles étaient associés à une augmentation de 40% de l’ensemble des coûts.

**CONCLUSION:**

Les directives OMS 2019 ont abouti à des modifications significatives du coût de traitement de la MDR-TB en Finlande et en Estonie. Ces modifications dépendent surtout du protocole et des pratiques de soins associées, avec des différences importantes entre pays et même à l’intérieur d’un pays en fonction des pratiques locales.

MULTIDRUG-RESISTANT TB (MDR-TB) increasingly challenges efforts to end the global TB epidemic.^1^ MDR-TB is diagnosed in a minority of TB patients, but accounts for disproportionally high resources in comparison with drug-susceptible TB (DS-TB).^2^ In 2019, a total of 206,030 cases of rifampicin-resistant and MDR-TB were diagnosed.^1^,^3^

In Finland, a country with median per capita gross domestic product (GDP) of $51,426 in 2019, reported TB incidence has steadily decreased over the past decades to 224 cases in 2019, representing 4.2 per 100 000 population. MDR-TB was detected in 1.7% of new cases.^4^–^6^ In Estonia too, an upcoming economy with a median per capita GDP of $38,915 in 2019, TB incidence has steadily decreased to 151 cases in 2019, a reported incidence of 11.1/100,000, with MDR- or RR-TB in 17% of new cases.^6^–^8^ In Russia, neighbouring to both Finland and Estonia, incidence is estimated at 50/100,000, with RR-TB in 35.0% of newly diagnosed TB patients.^6^

Until recently, treatment of MDR-TB consisted of long treatment courses including injectables, which were often poorly tolerated and had suboptimal results.^9^–^11^ Prolonged hospitalisation due to treatment with injectables was associated with high costs and psychological burden for the patient.^2^,^12^ Newly developed oral drugs bedaquiline (BDQ) and delamanid are associated with improved tolerability and efficacy.^13^ In 2019, the WHO adopted a new standardised regimen comprising only oral medications, including BDQ as a key drug, to be administered to all patients without contraindications.^14^

In the era of the injectables, several studies had evaluated costs of MDR-TB treatment in a variety of settings.^2^,^15^–^18^ Cost-effectiveness of the new BDQ-containing regimens was recently evaluated in several low TB burden countries.^19^,^20^ We hypothesise that costs of MDR-TB treatment may be differently affected by the new guidelines in high-income, low TB burden countries. In order to evaluate the impact of the 2019 WHO guidelines for MDR-TB on costs of MDR-TB treatment in Finland and Estonia, we obtained the standard treatment regimens according to national guidelines before and after adaptation to the new WHO guidelines and calculated the costs of drugs, as well as the costs related to monitoring and care in uncomplicated cases.

## METHODS

Standard regimens for the treatment of MDR-TB before and after adaptation to the 2019 WHO guidelines were obtained from Finland and Estonia. Costs of drugs, and care- and monitoring-related costs of these treatment regimens were obtained from Turku University Hospital, Turku, and Kuopio University Hospital, Kuopio, Finland and from Estonia. Costs of the standard DS-TB treatment were obtained as a control. Data were collected and analysed using Microsoft Excel (Microsoft, Redmond, WA, USA) and graphs were created using GraphPad Prism 9 (GraphPad Software, San Diego, CA, USA). As the study did not involve human subjects, ethical approval was not required.

### Sources and study sites

In Estonia, TB strategy is part of the Estonian Health Plan. The Estonian TB Register, located at the National Institute for Health Development (Tallinn, Estonia), comprises reports on all diagnosed cases of TB in Estonia, TB statistics and analysis of regimens prescribed, and efficiency of TB treatment. All MDR-TB patients are referred to the TB consilium, which consists of five pulmonologists from different hospitals and the head of the TB Register. As healthcare in Estonia is organised at the national level, regional comparisons (as was done in the case of Finland) was not possible.

In Finland, MDR-TB is reported to the Finnish Institute for Health and Welfare (Helsinki, Finland), and treatment of all MDR-TB cases is initiated and monitored by an expert group consisting of pulmonary disease specialists, infectious disease specialists, paediatricians and microbiologists, with all five university hospitals being represented. Two of the study authors (RP and TV) are members of this expert group and provided information on the regimens commonly used for MDR-TB in Finland and could thus used for this analysis. TB treatment is managed by governmental health institutions, and costs of medicines, healthcare of contacts and monitoring are similar in different institutions. However, regional organisation of healthcare may be different, and this may lead to differences in overall costs. For this study, we chose Turku University Hospital as our primary source of data on costs. Costs were also obtained from Kuopio University Hospital in order to check for possible regional differences in costs of MDR-TB treatment.

All drug dosages and other aspects of treatment and monitoring were calculated as for a hypothetical standard patient of 80 kg without comorbidities or complications, according to national guidelines and local practice. Supportive medicines to prevent or treat common side effects were left out of our calculations. All costs of hospitalisation, costs related to directly observed treatment (DOT), visits to the outpatient clinic, sick leave and other allowances paid to the patient were categorised under ‘care’. Costs of monitoring are based on national recommendations for clinical workup and regimen-specific monitoring.

In both countries, treatment of TB is provided free of charge for patients. Costs were obtained as charged for by suppliers, i.e., pharmacies and healthcare institutions, and as paid for by national health services.

## RESULTS

### MDR-TB treatment regimens in Estonia and Finland

Before the 2019 WHO guidelines, the preferred Estonian regimen for MDR-TB consisted of 18 months of treatment, including capreomycin (CPM) for the first 9 months (3 months 7 days a week, followed by 2 months 5 days a week, and then 4 months 3 days a week) (Estonian MDR-TB regimen 2016). Due to pyrazinamide (PZA) resistance in around 60% of cases, PZA was given only in a minority of cases, and was thus left out of our calculations.

After adaptation to the new guidelines, treatment of MDR-TB still included an injectable in 30–40% of cases due to frequency of and resistance profiles of MDR- and extensively drug-resistant TB (XDR-TB) cases. The duration of treatment is usually 14 months. BDQ is often given for 12 months (2 weeks 4 tablets 7 days a week, followed by 2 tablets 3 days a week for the rest of the treatment), depending on clinical aspects such as response to treatment and side effects. The Estonian 2019-A MDR-TB regimen consists of 14 months of treatment, including 12 months of BDQ and 6 months of the injectable drug, amikacin (AMK). In the most commonly used alternative regimen (MDR-TB regimen 2019-B), the injectable (AMK) is replaced by the oral drug prothionamide.

In Finland, before the 2019 WHO guidelines, the standard treatment regimen for MDR-TB lasted 20 months and included 6 months of AMK (Finnish 2016 MDR-TB regimen). After adaptation to the new guidelines, the standardised regimen now consists of 20 months treatment, including BDQ either for the full 20 months (MDR-TB regimen 2019-A) or for the first 6 months only (regimen MDR-TB 2019-B). Cycloserine (CS) and clofazimine (CFZ) are usually combined at the beginning of the treatment; either of these may be discontinued based on the patient’s resistance profile (typically available after 2 months) and possible side effects. For our analysis, we included CS and CFZ for the full duration of treatment. All standard treatment regimens for MDR-TB in Finland and Estonia are given in Table 1. WHO-approved standardised short regimens are not applied in Finland and Estonia.

**Table 1:** Standard MDR-TB regimens in this study according to national policy in Finland and in Estonia

	Estonia		Finland	
MDR-TB 2016^*^	CPM	First 9 months	AMK	First 6 months
	MFX	18 months	MFX	20 months
	LZD	18 months	LZD	20 months
	PTH	18 months	ETH	20 months
	CS	18 months	CS	20 months
			PZA	20 months
MDR-TB 2019-A^†^	BDQ	12 months	BDQ	20 months
	MFX	14 months	MFX	20 months
	LZD	14 months	LZD	20 months
	CFZ	14 months	CFZ	20 months
	CS	14 months	CS	20 months
	AMK	6 months		
MDR-TB 2019-B^‡^	BDQ	12 months	BDQ	First 6 months
	MFX	14 months	MFX	20 months
	LZD	14 months	LZD	20 months
	CFZ	14 months	CFZ	20 months
	CS	14 months	CS	20 months
	PTH	14 months		

^*^ Standard regimen for MDR-TB according to the 2016 WHO guidelines.

^†^ Standard regimen for MDR-TB as after adjustments to the 2019 WHO guidelines.

^‡^ Alternative standard regimen for MDR-TB as after adjustments to the 2019 WHO guidelines.

MDR-TB = multidrug-resistant TB; CPM = capreomycin; AMK = amikacin; MFX = moxifloxacin; LZD = linezolid; PTH = prothionamide; ETH = ethionamide; CS = cycloserine; PZA = pyrazinamide; BDQ = bedaquiline; CFZ = clofazimine.

### Costs of TB treatment in Estonia

**Figure 1:**
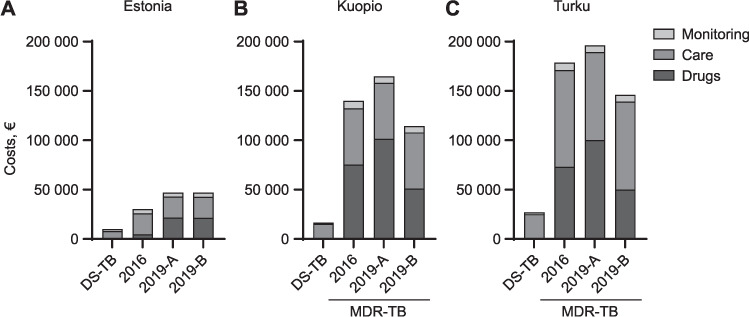
Costs of treatment of DS-TB and MDR-TB in Finland and Estonia. All costs in euros. DS-TB = drug-susceptible TB; MDR-TB = multidrug-resistant TB; MDR-TB 2016 = MDR-TB treated according to 2016 WHO guidelines; MDR-TB 2019-A and 2019-B = MDR-TB treatment regimen adjusted to the 2019 WHO guidelines.

In Estonia, drugs for the DS-TB treatment regimen cost €1.04/day during the intensive phase of treatment. All TB drug prices are shown in Table 2. According to the national guidelines, treatment of DS-TB can be started outpatient in eligible patients, in around 30% of cases. Although sick leave applies only to some patients, and related costs are highly variable, we estimated sick leave costs of €40/day for 4 months in patients treated for DS-TB. Overall costs of outpatient-treated DS-TB added up to €8,455. Drugs costs were €154 (2% of overall costs), while care and monitoring costs were respectively €6,176 (73%) and €2,126 (25%) (Figure 1). In case a month’s hospitalisation was required, overall costs went up to €11,907 due to an increase in care-related costs of up to €9,121 (80%). Monitoring costs are given in Table 3.

**Table 2:** Daily costs of drugs

	Estonia		Finland	
	
	Dose (mg)	Price (€)	Dose (mg)	Price (€)
Rifampicin	600	0.30	600	2.40
Isoniazid	300	0.07	300	1.19
Rifampicin-isoniazid	300 + 600	0.48	NA	NA
Ethambutol	1500	0.28	1500	4.96
Pyrazinamide	2000	0.28	2000	3.74
Amikacin	1000	4.30	1000	252.22
Capreomycin	1000	9.77	NA	NA
Bedaquiline	200	99.00	200	278.86
Linezolid	600	3.20	600	2.15
Moxifloxacin	400	0.96	400	0.77
Cycloserine	750	0.69	750	32.81
Clofazimine	200	4.00	100	1.97
Ethionamide	1000	NA	1000	28.34
Prothionamide	750	0.57	NA	NA

NA = not available.

**Table 3:** Costs related to monitoring during treatment of MDR-TB (all costs in euros)

	Finland			Estonia		
	
	DS-TB €	MDR-TB 2016^*^ €	MDR-TB 2019-A/B^†^ €	DS-TB €	MDR-TB 2016^*^ €	MDR-TB 2019-A/B^†^ €
Clinical microbiology^‡^	1157.00	3047.00	3047.00	1769.35	3433.95	3433.95
Other laboratory^§^	235.60	1581.79	1069.20	176.86	426.55	426.55
Radiology	434.00	776.00	776.00	127.04	184.16	184.16
Electrocardiogram	—	391.00	391.00	21.92	56.64	65.76
Ophthalmology	—	1600.00	1600.00	—	—	—
Audiogram	—	280	—	30.64	26.34	26.34
Total monitoring	1826.60	7675.79	6883.20	2125.81	4127.64	4136.76

^*^ Regimen before adaptation to 2019 WHO guidelines.

^†^ Regimens adapted to the 2019 WHO guidelines.

^‡^ Includes TB smear and culture, nucleic-acid amplification test, drug susceptibility testing and screening for HIV.

^§^ Includes chemistry, screening for 12s rRNA A1555G mutation, urine and serum drug levels.

MDR-TB = multidrug-resistant TB; DS-TB = drug-susceptible TB.

**Figure 2:**
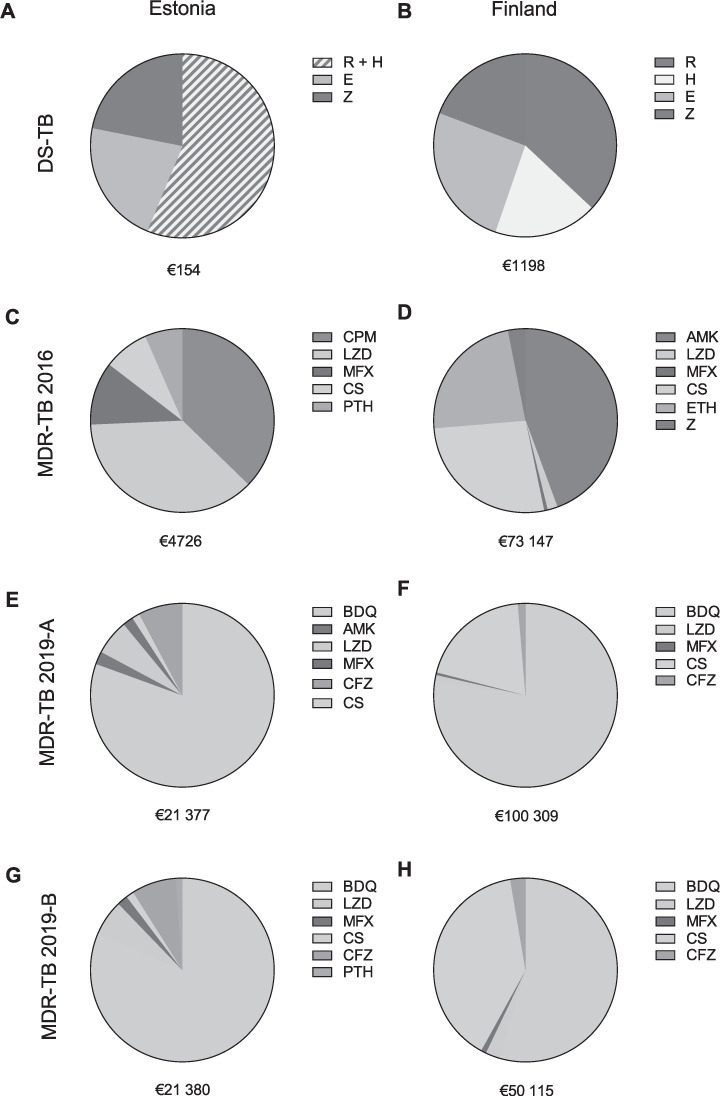
Relative contribution of individual drugs to total costs of drugs in TB. A, C, E and G: as in Estonia. B, D, F and H: as in Turku, Finland. A, B) DS-TB; C, D) treatment of MDR-TB in line with the 2016 WHO guidelines; E–H) preferred regimens of MDR-TB treatment (MDR-TB regimens 2019-A and 2019-B) adjusted according to the 2019 WHO guidelines. DS-TB = drug-susceptible TB; R = rifampicin; H = isoniazid; E = ethambutol; Z = pyrazinamide; MDR-TB 2016 = MDR-TB treated according to 2016 WHO guidelines; CPM = capreomycin; LZD = linezolid; MFX = moxifloxacin; CS = cycloserine; PTH = prothionamide; AMK = amikacin; ETH = ethionamide; MDR-TB 2019-A and 2019-B = MDR-TB treatment regimen adjusted to the 2019 WHO guidelines; BDQ = bedaquiline; CFZ = clofazimine; MDR-TB = multidrug-resistant TB.

Before 2019, MDR-TB required 4 months inhospital isolation in most cases in Estonia. Drug costs for the 2016 regimen were €4,726 (15% of overall costs). Costs for sick leave were estimated at €40/day for 8 months in patients treated for MDR-TB in Estonia. In total, €24,871 (74%) was required for care and €4,128 (14%) for monitoring, adding up to €33,664. CPM alone accounted for 37% (€1,739) of total drug costs, while linezolid (LZD) accounted for €1,728 (37%) in the 2016 regimen for MDR-TB in Estonia (Figure 2).

The Estonian MDR-TB treatment drug regimen 2019-A costs €21,631 and regimen 2019-B costs €21,380. In both regimens, BDQ accounted for €17,424. However, the new regimen is shorter and in-hospital isolation is usually required for only 2 months, resulting in a 14% decrease in care-related costs to €21,337 for the 2019 regimens. In the 2019 regimens, care accounted for 45% of overall costs in regimen 2019-A and 46% in regimen 2019-B in our calculations, while monitoring represented less than 10%. Overall costs, including drugs, care and monitoring, added up to €47,105 for regimen 2019-A and €46,854 for regimen 2019-B, an increase of respectively 40% and 39% in comparison with the 2016 regimen (Figures 1 and 2).

### Costs of TB treatment in Finland

In Finland, drugs for the DS-TB treatment regimen cost €12.29 during the intensive phase (Table 2). DS-TB treatment is commonly initiated in-hospital during a 2-week admission. Sick leave is typically prescribed for 2 months in patients with DS-TB. Overall costs of DS-TB treatment, including the first 2 weeks of treatment in-hospital, added up €26,859, of which €1,198 were for drugs (4%), €23,834 for care (89%) and €1,827 for monitoring (7%). Data are shown in Figure 1.

Overall costs of the 2016 MDR-TB treatment regimen, comprising 2 months of in-hospital treatment and 6 months of sick leave, added up to €178,714. Drugs alone costed €73,147 (41%), while care and monitoring costs were respectively €97,891 (55%) and €7,675 (4%). AMK made up 44% (€32,452) of total drug costs, followed by CS (27%, €19,688) and ethionamide (23%, €17,002) (Figure 2).

Costs of Finnish MDR-TB treatment regimen 2019-A (including 20 months of BDQ) added up to a total of €196,213, which is €17,498 (10%) more than the old regimen. Drug costs accounted for €100,309 (51%), while care and monitoring costs were respectively €89,020 (45%) and €6,883 (4%). When BDQ was given only for the first 6 months (regimen 2019-B), drug costs significantly dropped to €50,115 (i.e., 34% of the overall costs of €146,018) in Turku, Finland.

In Turku, DOT is organised at home 5 days a week; this costed €11,700 for the duration of the entire DS-TB treatment (49% of care-related costs), €50,991 (52%) for the 2016 MDR-TB regimen and €42,120 (47%) for the 2019 regimen. Hospitalisation in isolation costed €8,400 for 2 weeks in DS-TB (35% of costs of care) and €36,000 for 2 months in MDR-TB patients. However, in Kuopio, the second Finnish site of this study, DOT was performed at a local health centre due to lack of local resources. Costs of DOT visits (€100/day) were replaced by costs of visit at the local healthcare centre, which was only €17/visit; this led to significantly lower care-related costs (€14,158 for DS-TB in Kuopio vs. €23,834 in Turku and €56,854 for the new MDR-TB regimens vs. €89,020 in Turku; Figure 1).

## DISCUSSION

In Estonia, a high MDR-TB burden country, the adaptation to the guidelines led to a more than fourfold increase in drug costs and around 40% rise in overall costs of MDR-TB treatment, mainly driven by the introduction of BDQ. In Finland, the adaptation of MDR-TB treatment to the 2019 WHO guidelines led to a 10% increase (regimen 2019-A) or an 18% decrease (regimen 2019-B) of overall costs in uncomplicated cases, depending on the duration of BDQ treatment, 6 months or 20 months, respectively. While the generic price of BDQ was estimated at US$8–$17 per month, the high costs of the drug are due to the Johnson & Johnson patent, although the drug is increasingly being made available at reduced prices in low- and middle-income countries.^21^

In Finland, other drugs with a considerable impact on total costs are AMK, ethionamide and CS in the 2016 MDR-TB regimen and CS in the 2019 MDR-TB regimens. Changes in costs related to care and monitoring are of minor importance in comparison to changes in drug-related costs.

This study had several limitations. First, resistance profiles were highly variable and treatment of MDR-TB was rarely uncomplicated. Treatment regimens including BDQ may lead to fewer side effects and better treatment results.^13^ Therefore, costs as estimated in this study may differ from real-life costs of MDR-TB treatment. For instance, prolonged treatment with LZD and CS are associated with side effects. Individual adjustments to the regimens may lead to important changes in real-life costs. Real-life treatment costs may also be affected by comorbidities and comedications. However, as regimen changes have been introduced recently, analysis of changes in real-life costs will be available only after several years.^22^

Second, additional costs to the patient, such as loss of income not covered by sick leave, can vary widely and are not accounted for in the present study.^23^

Third, in our data, changes in costs may depend on national guidelines and local practices and prices of drugs may vary considerably between countries, which is in accordance with findings from previous studies.^23^ Several TB drugs are provided by pharmaceutical companies at different prices depending on the economic status of the country. Furthermore, WHO and The Global Fund provide support for MDR-TB treatment to some countries. Therefore, the results of this study cannot be generalised.

The WHO recommends treatment initiation in outpatient settings. In both Finland and Estonia, hospitalisation is still common in the first months of treatment. In pulmonary TB, hospitalisation often requires isolation, which can be costly. Treatment initiation in outpatient settings could considerably reduce costs, and this is especially of relevance in high TB burden countries. Care-related costs may be further reduced by replacing DOT with video-observed therapy (VOT), which is up to now being used only in a selection of patients in both countries. In countries where cost of MDR-TB treatment placed a high burden on society, increased costs may lead to reluctance to adapt treatment regimens to the 2019 WHO guidelines. Any effort to reduce costs of MDR-TB treatment should be carefully weighed against possible effects on accessibility, effectiveness and safety of treatment.

In conclusion, national adaptations of the 2019 WHO guidelines have led to significant changes in the costs of MDR-TB treatment in Finland and Estonia. These changes depend on the drug regimen used, with BDQ as the main driver of costs, and on care-related practice, with important differences between countries and even within the same country.

The datasets used and/or analysed during the current study are available from the corresponding author on reasonable request.
